# mTOR Hyperactivation by Ablation of Tuberous Sclerosis Complex 2 in the Mouse Heart Induces Cardiac Dysfunction with the Increased Number of Small Mitochondria Mediated through the Down-Regulation of Autophagy

**DOI:** 10.1371/journal.pone.0152628

**Published:** 2016-03-29

**Authors:** Manabu Taneike, Kazuhiko Nishida, Shigemiki Omiya, Elham Zarrinpashneh, Tomofumi Misaka, Rika Kitazume-Taneike, Ruth Austin, Minoru Takaoka, Osamu Yamaguchi, Michael J. Gambello, Ajay M. Shah, Kinya Otsu

**Affiliations:** 1 Cardiovascular Division, King’s College London British Heart Foundation Centre of Excellence, London, United Kingdom; 2 Department of Cardiovascular Medicine, Graduate School of Medicine, Osaka University, Suita, Osaka, Japan; 3 Division of Medical Genetics, Emory University School of Medicine, Atlanta, Georgia, United States of America; University of Alabama at Birmingham, UNITED STATES

## Abstract

Mammalian target of rapamycin complex 1 (mTORC1) is a key regulator of cell growth, proliferation and metabolism. mTORC1 regulates protein synthesis positively and autophagy negatively. Autophagy is a major system to manage bulk degradation and recycling of cytoplasmic components and organelles. Tuberous sclerosis complex (TSC) 1 and 2 form a heterodimeric complex and inactivate Ras homolog enriched in brain, resulting in inhibition of mTORC1. Here, we investigated the effects of hyperactivation of mTORC1 on cardiac function and structure using cardiac-specific TSC2-deficient (*TSC2*^-/-^) mice. *TSC2*^-/-^ mice were born normally at the expected Mendelian ratio. However, the median life span of *TSC2*^-/-^ mice was approximately 10 months and significantly shorter than that of control mice. *TSC2*^-/-^ mice showed cardiac dysfunction and cardiomyocyte hypertrophy without considerable fibrosis, cell infiltration or apoptotic cardiomyocyte death. Ultrastructural analysis of *TSC2*^-/-^ hearts revealed misalignment, aggregation and a decrease in the size and an increase in the number of mitochondria, but the mitochondrial function was maintained. Autophagic flux was inhibited, while the phosphorylation level of S6 or eukaryotic initiation factor 4E -binding protein 1, downstream of mTORC1, was increased. The upregulation of autophagic flux by trehalose treatment attenuated the cardiac phenotypes such as cardiac dysfunction and structural abnormalities of mitochondria in *TSC2*^-/-^ hearts. The results suggest that autophagy via the TSC2-mTORC1 signaling pathway plays an important role in maintenance of cardiac function and mitochondrial quantity and size in the heart and could be a therapeutic target to maintain mitochondrial homeostasis in failing hearts.

## Introduction

Mammalian target of rapamycin complex 1 (mTORC1) plays a critical role in the regulation of cell growth, proliferation and metabolism [1]. mTORC1 positively controls protein synthesis by phosphorylating downstream substrates, namely eukaryotic initiation factor 4E (eIF4E)-binding protein 1 (4E-BP1) and p70 ribosomal S6 kinase 1 (S6K1). The phosphorylation of 4E-BP1 prevents its binding to eIF4E, enabling eIF4E to promote cap-dependent translation [2]. Active S6K1 phosphorylates several substrates, including S6, that function in translation initiation as well as other steps that drive protein production. In addition to protein synthesis, mTORC1 positively controls mitochondrial function through a yin yang 1-peroxisome proliferator activated receptor coactivator 1alpha (PGC-1alpha) transcriptional complex [3].

On the other hand, mTORC1 also plays an important role in protein degradation. In mammalian cells, there are two major systems of protein degradation, namely the ubiquitin/proteasome system and autophagy. mTORC1 inhibits autophagy [4] by phosphorylating unc-51 like autophagy activating kinase 1 (ULK1), a mammalian ortholog of Atg1 [5]. In autophagy, an isolation membrane sequesters a part of the cytoplasm or organelles including mitochondria to form an autophagosome [6]. The autophagosome fuses with a lysosome and becomes an autolysosome. Then, the materials contained in the autolysosome are degraded by lysosomal enzymes. Autophagy contributes to macromolecule synthesis and energy production as well as quality control of cytoplasmic proteins and organelles. We have previously reported that constitutive autophagy in the heart is a homeostatic mechanism for maintaining cardiomyocyte size, global cardiac structure and function and the quality of mitochondria [7].

Mitochondria are important organelles for ATP production and dynamic organelles. They continuously undergo fusion and fission during cell life, exhibiting short round-shaped or elongated morphology. Autophagy will be preceded by mitochondrial fission, which divides elongated mitochondria into pieces of manageable size for engulfment by isolation membrane. When autophagy is induced in the cells by nutrient depletion, mitochondria elongate [8]. Elongated mitochondria are spared from autophagy and maintain ATP production upon starvation.

mTORC1 is positively regulated by Ras homolog enriched in brain (Rheb). Rheb is a small GTPase, that exists either in an active GTP-bound or an inactive GDP-bound state [9]. In the signaling pathway towards mTORC1, tuberous sclerosis complex (TSC) 1 and 2 exist upstream of Rheb. Mutations in either the *TSC1* or *TSC2* gene are the principal cause of TSC, which is an autosomal dominant multisystemic disorder characterized by the development of numerous benign tumours (e.g. hamartomas) in many organs, such as brain, kidneys, skin, heart and lungs [10]. There is no homology between the 140 kDa TSC1 and the 200 kDa TSC2 proteins. TSC1 and TSC2 associate with each other to form a heterodimeric complex as a regulatory unit and a catalytic unit, respectively. The TSC1/2 complex is a GTPase-activating protein that catalyses the conversion of Rheb-GTP to Rheb-GDP [11] and inactivates Rheb, resulting in inhibition of mTORC1 signaling [12, 13]. Akt-mediated phosphorylation of TSC2 inhibits the function of the TSC1/2 complex [14], resulting in activation of mTORC1. However, the existence of an mTORC1-dependent feedback mechanism blocks growth-factor-stimulated phosphorylation of Akt [15].

Studies using *Drosophila* revealed that loss-of-function mutations of *TSC1*, *TSC2* and both genes combined lead to organ overgrowth via increased cell proliferation and cell-autonomous increase in size [16, 17]. In mice, the deficiency of mTORC1 leads to embryonic lethality [18, 19]. Cardiac-specific mTOR-deficient mice are also embryonic lethal [20]. Furthermore, ablation of *Mtor* in the adult mouse heart results in a fatal, dilated cardiomyopathy, which is mainly caused by enhanced dephosphorylation of 4E-BP1 [21]. These data suggest that mTOR has a critical role in protein synthesis to maintain cardiac function.

To investigate the role of the mTORC1 signaling pathway in the heart, mouse models where its activity is altered by modulation of upstream signaling molecules will be more physiologically relevant than *Mtor* ablation model. We previously investigated the role of mTORC1 in the heart using cardiac-specific Rheb-deficient mice, where mTORC1 was downregulated [22]. We found that mTORC1 activity through Rheb is essential for normal cardiac hypertrophic growth during the postnatal period, although autophagy was not related to the phenotypes observed in Rheb-deficient hearts. Since conventional TSC2-deficient mice are embryonic lethal [23, 24], we used cardiac-specific TSC2-deficient mice to examine the effect of mTORC1 hyperactivation on protein degradation and mitochondrial dynamics as well as protein synthesis in cardiomyocytes in this study. The ablation of *TSC2* in mouse hearts leads to Rheb activation, mTORC1 activation, ULK1 phosphorylation and then finally inhibition of autophagy. Our results suggest that autophagy via the TSC-mTORC1 signaling pathway plays an important role in determining the size and number of mitochondria to maintain cardiac function.

## Materials and Methods

### Animals

All *in vivo* procedures in this study were carried out in accordance with the Guidance on the Operation of the Animals (Scientific Procedures) Act, 1986 (UK Home Office) or the Guidelines for Animal Experiments of Osaka University and the Japanese Act on Welfare and Management of Animals (No. 105). King’s College London Ethical Review Process Committee and UK Home Office (Project Licence No. PPL70/7260) or Osaka University Animal Experimentation Committee approved this study. Mortality was described as a possibility in the approved study protocols. Mice were given food and water *ad libitum*. Mice were monitored daily during the animal study including the survival study to minimize suffering. Animals that showed signs of significant clinical cardio-respiratory distress due to the development of heart failure (e.g., increased respiratory rates, reduced activity, piloerection, hunched posture) were immediately euthanized by CO_2_ exposure. In a small pilot study approved by Osaka University Animal Experimentation Committee and performed under the Guidelines for Animal Experiments of Osaka University and the Japanese Act on Welfare and Management of Animals (No. 105), 4 mice showed unexpected death (S1 Protocol). Autopsy of all the dead mice showed pleural effusion and increased lung weight, suggesting the cause of death was heart failure. Thus, euthanized mice were considered as deaths. All the mice were harvested in the early morning. We didn’t detect any difference in food consumption and body weight gaining among groups used in this study.

### Generation of cardiac-specific TSC2-deficient mice and echocardiography

The generation of mice bearing a *TSC2*^flox^ allele, in which exon 2–4 of the *TSC2* gene is flanked by two *loxP* sequences, has been previously reported [25]. Mice bearing the *TSC2*^flox^ allele were crossed with transgenic mice expressing *Cre* recombinase under the control of alpha-myosin heavy chain promoter (alpha-MyHC-*Cre*) [26]. The genetic backgrounds of the *TSC2*^flox/flox^ and alpha-MyHC-*Cre* mice are 129/SvJ x C57B/6J and C57B/6J, respectively. All mice which were used in this study were male. Vevo 2100 system with a 22–55-MHz linear transducer (Visual Sonics) was used to perform echocardiography on awake or anaesthetized mice. The echocardiography was performed on awake mice, those were trained prior to the actual measurement. Isoflurane was used for echocardiography on anesthetized mice. The sonographer blinded to the groups. Trans-thoracic M-mode images were acquired in parasternal short axis view. Fractional shortening (FS) and end-diastolic left ventricular (LV) mass were calculated as 100 x (end-diastolic LV internal dimension (LVIDd)—end-systolic LV internal dimension (LVIDs))/LVIDd and 1.05 x [(LVIDd + end-diastolic interventricular septal thickness (IVSd) + LV posterior wall thickness (LVPWd))^3^ - (LVIDd)^3^], respectively.

### Antibodies

Specific antibodies targeted to the following proteins were used for the Western blot analysis in 1,000 times dilution (except for the one to alpha-tubulin (3,000)) and according to manufacturers’ instructions: TSC1 (rabbit monoclonal, Cell Signaling Technology (CST), #6935), TSC2 (rabbit monoclonal, CST, #4308), phosphorylated Akt (rabbit monoclonal, CST, #4058), Akt (rabbit polyclonal, CST, #9272), phosphorylated AMP-activated protein kinase (AMPK) (rabbit monoclonal, CST, #2535), AMPK (rabbit polyclonal, CST, #2532), phosphorylated S6 (rabbit, polyclonal, CST, #2215), S6 (rabbit monoclonal, CST, #2217), ubiquitin (rabbit polyclonal, CST, #3933), microtubule-associated protein 1 light chain 3 (LC3) B (rabbit polyclonal, CST, #2775), alpha-tubulin (mouse monoclonal, CST, #3873), voltage-dependent anion channel (VDAC) (rabbit polyclonal, CST, #4866), Parkin (rabbit polyclonal, CST, #2132), 4E-BP1 (rabbit polyclonal, Abcam, ab2606), PINK1 (rabbit polyclonal, Abcam, ab23707), KDEL proteins (mouse monoclonal, Enzo Life Sciences, ADI-SPA-827), sequestosome 1/p62 (p62) (C-terminal specific) (guinea pig polyclonal, Progen, GP62-C), translocase of inner mitochondrial membrane 23 homolog (Timm23) (rabbit polyclonal, Proteintech, 11123-1-AP), 4-hydroxy-2-nonenal michael adducts (HNE) (rabbit polyclonal, Calbiochem, 393207). Tissues were lysed in homogenization buffer (50 mM Tris-HCl, pH 7.4, 150 mM NaCl, 1 mM EDTA, 1 mM EGTA, 2.5 mM Na-orthovanadate, 2.5 mM Na-pyrophosphate, 1 mM β-glycerophosphate, 1% Triton X-100) with protease inhibitors (PMSF or protease inhibitor cocktail (Sigma)). Western blots were incubated with the secondary antibodies, followed by developing with an infrared imaging system, ODYSSEY CLx (LI-COR). NIH Image J software (version 1.46r) or Image Studio software (LI-COR) was used to perform densitometric analyses.

### Histological analysis

The heart was excised and immediately fixed in buffered 4% paraformaldehyde in PBS, embedded in paraffin, and sectioned to a thickness of 6 micrometers. Haematoxylin and eosin (H&E) staining was performed on serial sections. For wheat germ agglutinin (WGA) staining, heart samples were mounted and frozen in O.C.T. compound (Thermo Scientific), cryo-sectioned to a thickness of 6 micrometers and stained with FITC-conjugated lectin (Sigma) to measure cross sectional area of cardiomyocytes. To determine the number of cells undergoing nuclear fragmentation, TdT-mediated dUTP-biotin nick end labeling (TUNEL) assay was performed on paraffin-embedded heart sections, using *In situ* Apoptosis Detection Kit (Takara Bio Inc.). For electron microscopy, the heart was perfused and then fixed with 2% formaldehyde and 2% glutaraldehyde. LV tissues were processed for transmission electron microscopy. We measured the area of mitochondria or counted the number of mitochondria in micrographs taken at 10,000-fold magnification, using NIH Image J software (version 1.46r) for 4 fields per mouse.

### Quantitative real-time RT-PCR

We isolated total RNA from the LV using the TRIzol reagent (Life Technologies). We determined mRNA expression levels for atrial natriuretic factor (*Nppa*), beta-myosin heavy chain (*Myh7*), collagen type 1 (*Col1a2*), *Pgc-1a* and *Gapdh* by quantitative RT-PCR. For reverse transcription, we used SuperScript II Reverse Transcriptase (Life Technologies). Real-time PCR reaction was performed using Power SYBR Green Master Mix (Life Technologies) and PCR primers designed as follows: forward 5’-TCGTCTTGGCCTTTTGGCT-3’ and reverse 5’-TCCAGGTGGTCTAGCAGGTTCT-3’ for *Nppa*, forward 5’-ATGTGCCGGACCTTGGAAG-3’ and reverse 5’-CCTCGGGTTAGCTGAGAGATCA-3’ for *Myh7*, forward 5’-ACGCGGACTCTGTTGCTGCT-3’ and reverse 5’-GCGGGACCCCTTTGTCCACG-3’ for *Col1a2*, forward 5’-AATCAGACCTGACACAACGC-3’ and reverse 5’-GCATTCCTCAATTTCACCAA-3’ for *Pgc-1a* and forward 5’-ATGACAACTTTGTCAAGCTCATTT-3’ and reverse 5’-GGTCCACCACCCTGTTGCT-3’ for *Gapdh*. We constructed real-time PCR standard curves using the corresponding complementary DNA. All data were normalized to *Gapdh* content and are expressed as fold increase over the control group.

### Mitochondrial enzyme activities

The activities of NADH cytochrome-*c* oxidoreductase (complex I + III) and succinate cytochrome-*c* oxidoreductase (complex II + III) were determined in mitochondrial fractions freshly isolated from hearts using previously described spectrophotometric methods [27]. Results are shown as nmol/min/mg protein.

### Tissue ATP concentration and the ratio of ADP to ATP

The heart was excised and immediately frozen in liquid nitrogen. The samples were ground using a pestle and mortar chilled with liquid nitrogen. ATP concentration and the ratio of ADP to ATP were measured using EnzyLight ADP/ATP Ratio Assay Kit (BioAssay Systems).

### Trehalose treatment

To examine the role of autophagy in the development of heart failure in *TSC2*^-/-^ mice, the mice were treated with 1% trehalose in drinking water for 10 weeks starting from 6 weeks after birth. The water solutions were changed twice weekly.

### Statistical analysis

Results are shown as the mean ± S.E.M. Paired data were evaluated using a Student’s *t*-test. The Kaplan-Meier method with Logrank test was used for survival analysis. Distribution of mitochondrial size was analysed using Chi-square or Mann-Whitney U test. A value of *P* < 0.05 was considered statistically significant.

## Results

### Generation and characterization of mice lacking TSC2 in the heart

**Fig 1:**
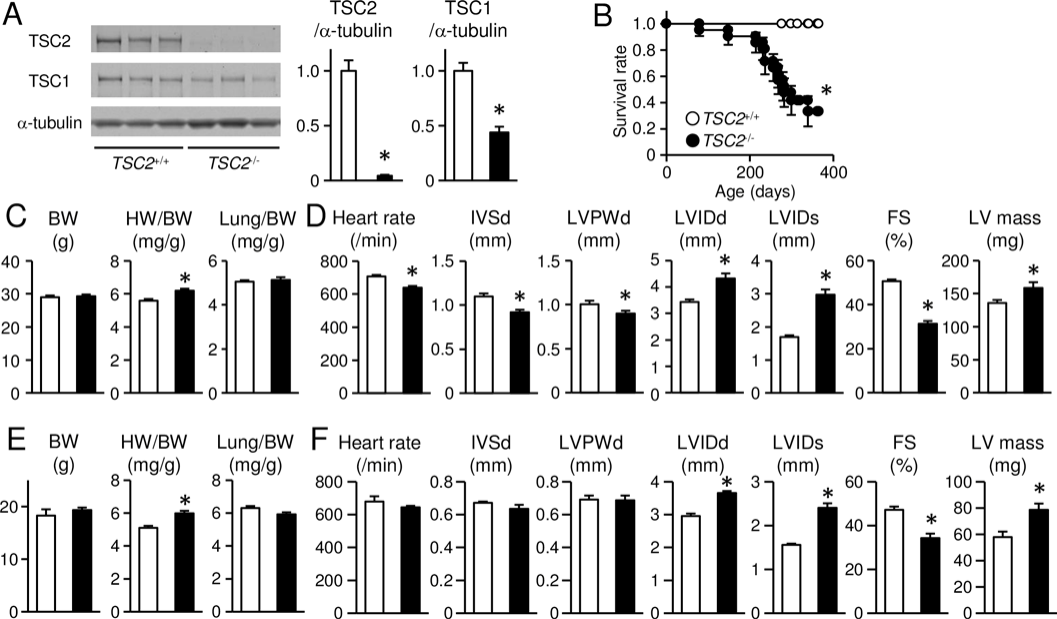
Development of cardiac dysfunction in cardiac-specific TSC2-deficient mice. (A): Western blot analysis of TSC proteins in adult cardiomyocytes isolated from 8-week-old *TSC2*^+/+^ and *TSC2*^-/-^ mice. (B): Survival curve of the mice. (C): Physiological analysis of the mice at 4 months of age. BW, HW/BW, and Lung/BW indicate body weight, whole heart-to-body-weight ratio, and lung-to-body-weight ratio, respectively. (D): Echocardiographic analysis of the awake mice at 4 months of age. (E): Physiological analysis of the mice at 4 weeks of age. (F): Echocardiographic analysis of the awake mice at 4 weeks of age. Open and closed bars represent *TSC2*^+/+^ and *TSC2*^-/-^ mice, respectively. Values represent the mean ± S.E.M. of data from 5–6 samples in (A), from 20 mice in (B), from 7–8 mice in (C), from 9 mice in (D), from 5–7 mice in (E) and from 3–5 mice in (F) in each group. *P < 0.05 versus corresponding control.

**Fig 2:**
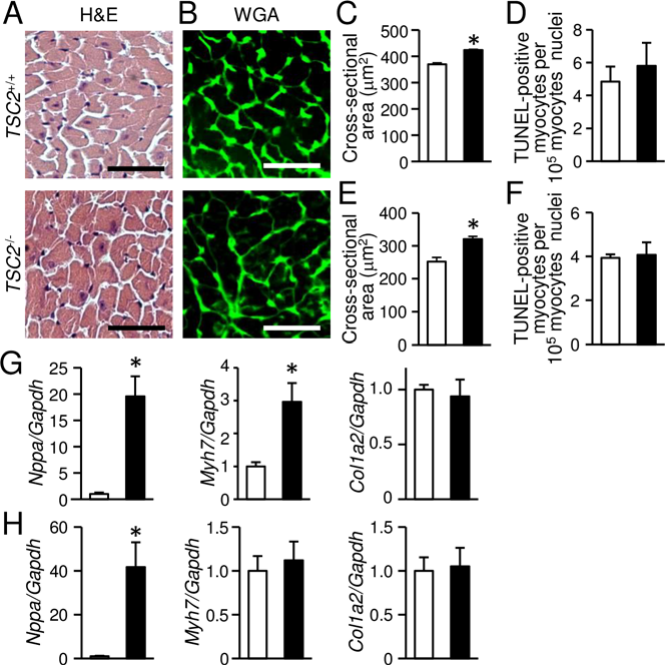
Development of cardiac hypertrophy in *TSC2*^-/-^ mice. The data at 4 months of age in (A) to (C), (E) and (G). The data at 4 weeks of age in (D), (F) and (H). (A): Haematoxylin and eosin (H&E)-stained heart sections. (B): Wheat germ agglutinin (WGA)-stained heart sections. (C) and (D): Cardiomyocyte cross-sectional area was measured by tracing the outline of 100 myocytes in each section. (E) and (F): Number of TUNEL-positive cardiomyocytes. (G) and (H): mRNA expressions of *Nppa*, *Myh7* and *Col1a2* were determined by quantitative RT-PCR. Data were normalized to the *Gapdh* content and are expressed as fold increase over levels in the *TSC2*^+/+^ group. Open and closed bars represent *TSC2*^+/+^ and *TSC2*^-/-^ mice, respectively. Values represent the mean ± S.E.M. of data from 4 mice in (C) and (E), from 3 mice in (D) and (F), from 3–4 mice in (G) and from 4–5 mice in (H) in each group. *P < 0.05 versus control group. Scale bars, 50 micrometers.

To investigate the effect of the upregulated mTORC1 signaling pathway in the heart, we generated cardiac-specific TSC2-deficient mice. *TSC2*^flox/flox^ mice were crossed with mice expressing alpha-myosin heavy chain promoter driven *Cre* recombinase (alpha-MyHC-*Cre*) to obtain *TSC2*^flox/flox^;alpha-MyHC-*Cre*^+^ (*TSC2*^-/-^) mice. *TSC2*^flox/flox^;alpha-MyHC-*Cre*^-^ (*TSC2*^+/+^) littermates were used as controls. The alpha-MyHC-*Cre*^+^ mice exhibited similar cardiac function to their control non-transgenic mice up to 12 months of age [28] and had normal life span. Western blot analysis revealed a 95% reduction of TSC2 protein in cardiomyocytes isolated from 8-week-old *TSC2*^-/-^ hearts compared with those from *TSC2*^+/+^ hearts (Fig 1A). There was significant reduction in the expression level of TSC1 protein in *TSC2*^-/-^ hearts. TSC1 and TSC2 associate with each other to form a heterodimer [29, 30]. Thus, there is a possibility that the stability of TSC1 is reduced by absence of TSC2. *TSC2*^-/-^ mice were born normally at the expected Mendelian ratio (*TSC2*^-/-^:*TSC2*^+/+^ = 38:43 delivered from crossing *TSC2*^-/-^ with *TSC2*^+/+^). However, *TSC2*^-/-^ mice began to die at 4 months of age, and the median life span was approximately 10 months, which was significantly shorter than that of *TSC2*^+/+^ mice (Fig 1B). The mortality at 4 months was 5%. Autopsy of dead mice showed pleural effusion and increased lung weight, suggesting the cause for death was heart failure. We evaluated physiological parameters in 4-month-old *TSC2*^-/-^ mice (Fig 1C). There was no significant difference in body weight or the ratio of lung to body weight between *TSC2*^-/-^ and *TSC2*^+/+^ mice. The ratio of whole heart to body weight was significantly higher in *TSC2*^-/-^ mice. Then, we performed trans-thoracic M-mode echocardiography on awake *TSC2*^-/-^ mice to evaluate cardiac morphology and function (Fig 1D). Heart rate (HR), IVSd and LVPWd were significantly reduced and LVIDd and LVIDs were significantly increased in *TSC2*^-/-^ mice compared with those in *TSC2*^+/+^ mice. Furthermore, FS, an indicator of cardiac systolic function, was significantly lower and calculated LV mass was significantly increased in *TSC2*^-/-^ mice compared with those in *TSC2*^+/+^ mice. Since there was a possibility that reduced HR was the cause for reduction of systolic function in *TSC2*^-/-^ mice, we performed echocardiography on anaesthetized mice to obtain the data under the same HR (S1 Fig). Under the situation, *TSC2*^-/-^ mice showed increase in LV chamber size and decrease in LV contractility. Histological analysis by H&E staining indicated no significant fibrosis or cell infiltration (Fig 2A). WGA staining showed a significant increase in the cross-sectional area of cardiomyocytes in *TSC2*^-/-^ mice compared with that in *TSC2*^+/+^ mice (Fig 2B and 2C). There was no significant difference in number of TUNEL-positive cardiomyocytes between the two groups (Fig 2E). Quantitative RT-PCR analyses showed higher mRNA expression levels of *Nppa* and *Myh7*, markers for cardiac remodeling, but not that of *Col1a2*, a marker for fibrosis, in *TSC2*^-/-^ hearts (Fig 2G) in agreement with the histological analyses. These data indicate that *TSC2*^-/-^ mice showed cardiomyocyte hypertrophy, LV chamber dilatation and cardiac dysfunction without considerable fibrosis, pulmonary congestion or apoptotic cardiomyocyte death at 4 months of age.

### Biochemical features related to the mTORC1 signaling pathway in 4-month-old *TSC2^-/-^* mice

**Fig 3:**
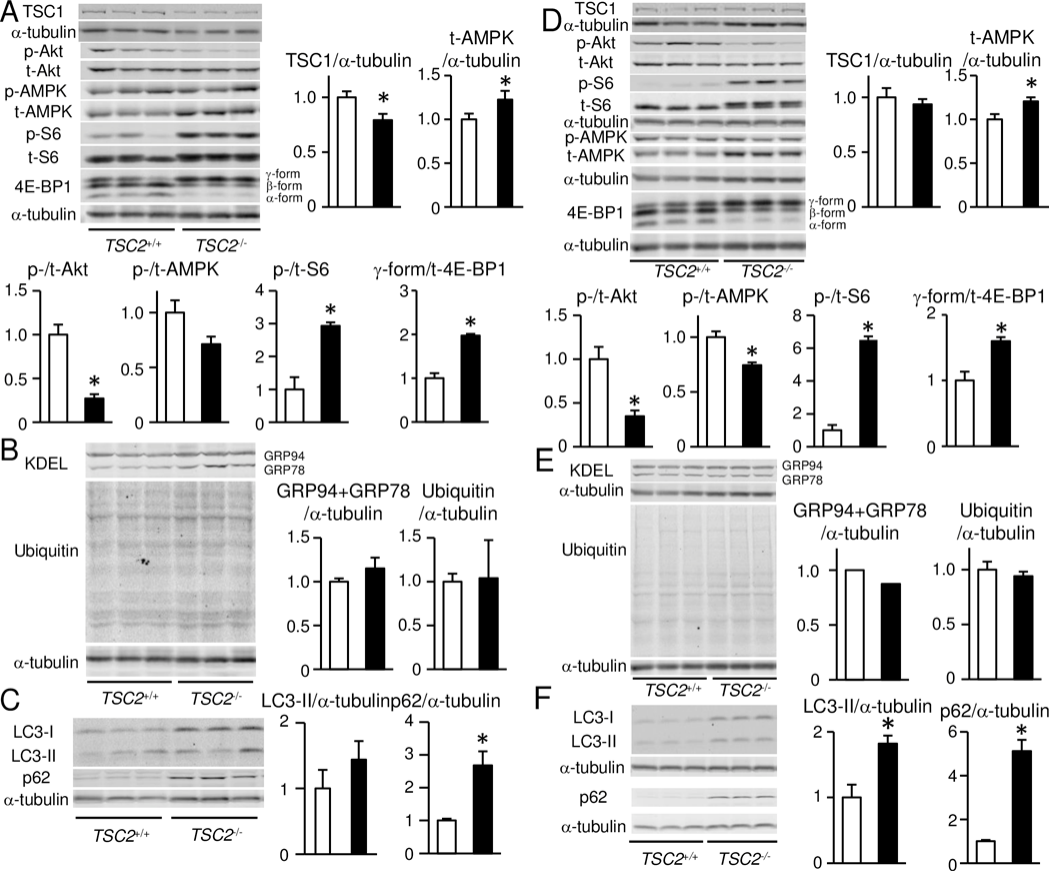
Upregulation of the mTORC1 signaling pathway in *TSC2*^-/-^ hearts. The data at 4 months of age in (A) to (C). The data at 4 weeks of age in (D) to (F). (A) and (D): Western blot analysis of signaling proteins upstream or downstream of mTORC1 in the heart of *TSC2*^+/+^ or *TSC2*^-/-^ mice. p-Akt, t-Akt, p-AMPK, t-AMPK, p-S6 and t-S6 indicate phosphorylated Akt, total Akt, phosphorylated AMPK, total AMPK, phosphorylated S6 and total S6, respectively. Data of phosphorylated proteins were normalized to corresponding total protein content, TSC1 and t-AMPK to alpha-tubulin and gamma-form to total 4E-BP1 (t-4E-BP1), respectively. (B) and (E): Western blot analyses of KDEL and ubiquitinated proteins. (C) and (F): Western blot analyses of LC3 and p62. Data were normalized to the alpha-tubulin protein. All data are expressed as fold increase over levels in the *TSC2*^+/+^ group. Open and closed bars represent *TSC2*^+/+^ and *TSC2*^-/-^ mice, respectively. Values represent the mean ± S.E.M. of data from 3–7 mice in each group. *P < 0.05 versus corresponding control.

We examined the effect of *TSC2* ablation on the mTORC1 signaling pathway in 4-month-old mouse hearts (Fig 3A). TSC1 was significantly decreased in *TSC2*^-/-^ hearts although the difference was smaller compared to that in isolated cardiomyocytes. There were significant increases in the phosphorylation levels of S6 and 4E-BP1 in *TSC2*^-/-^ hearts compared with *TSC2*^+/+^ hearts, indicating the activation of mTORC1 and resultant upregulation of protein synthesis in *TSC2*^-/-^ hearts. The phosphorylation level of Akt was significantly decreased in *TSC2*^-/-^ hearts compared with *TSC2*^+/+^ hearts, which is consistent with the existence of a negative feedback loop [31]. AMPK is a positive regulator of TSC2 protein, and its activation in response to a decrease in ATP and an increase in AMP in cytosol leads to TSC2 phosphorylation [1]. The phosphorylation level of AMPK was significantly decreased in *TSC2*^-/-^ group although the expression level of AMPK was increased. To evaluate the accumulation of ubiquitinated, misfolded or unnecessary proteins due to the excess of protein synthesis, we performed Western blot analyses for ubiquitin and KDEL proteins (GRP78 and GRP94), markers for endoplasmic reticulum stress (Fig 3B). However, we detected no significant differences in the expression levels of the proteins between the two groups. Since mTORC1 is known to be a negative regulator of autophagy in mammalian cells [4], we estimated the autophagic activity in *TSC2*^-/-^ hearts (Fig 3C) [32]. The conversion of LC3-I to LC3-II is an essential step for autophagosome formation. The expression level of LC3-II was not significantly different between the two groups. However, p62, a marker for autophagic flux, was significantly accumulated in *TSC2*^-/-^ hearts compared to *TSC2*^+/+^ hearts.

### Mitochondrial morphology in 4-month-old *TSC2^-/-^* hearts

**Fig 4:**
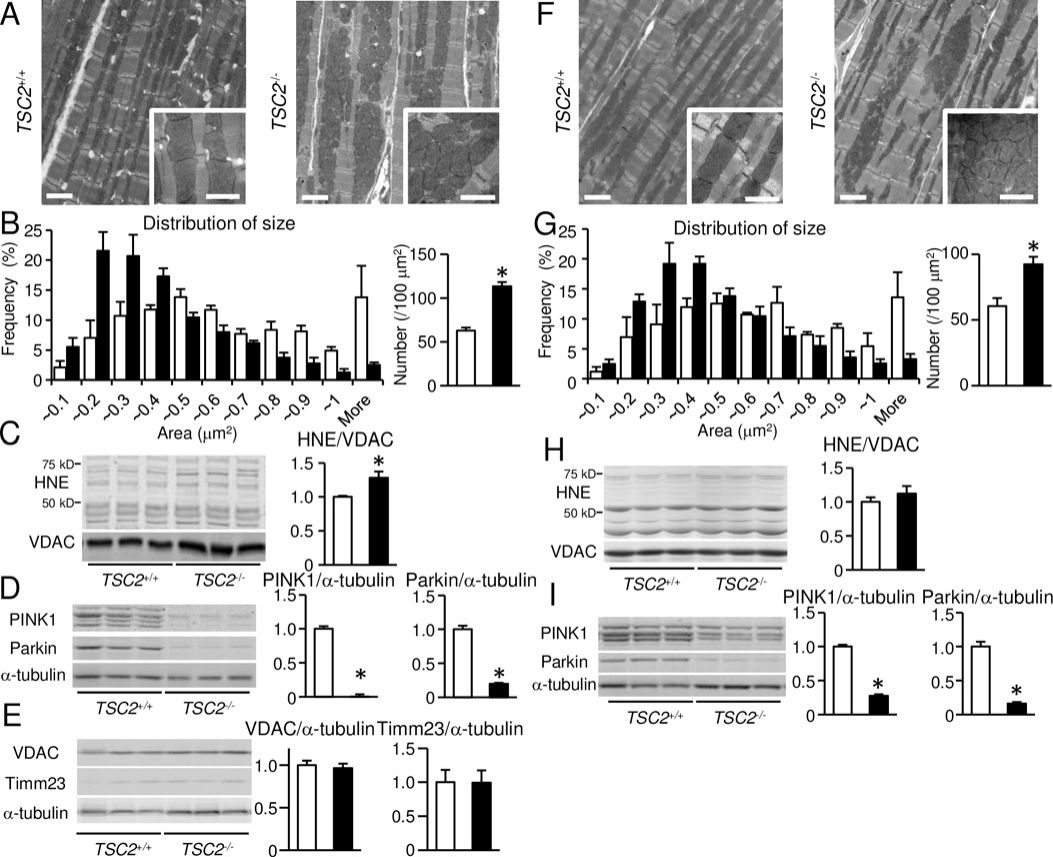
Morphological abnormality in mitochondria of *TSC2*^-/-^ hearts. The data at 4 months of age in (A) to (E). The data at 4 weeks of age in (F) to (I). (A) and (F): Electron micrographs of *TSC2*^+/+^ and *TSC2*^-/-^ mouse hearts. Intramitochondrial structure is shown in the insets. (B) and (G): Quantitative analysis of mitochondria in size and number. (C) and (H): Western blot analysis of HNE proteins in the mitochondrial fraction isolated from the heart. VDAC is used as loading control. (D) and (I): Western blot analysis of proteins related to mitophagy. (E): Western blot analysis for mitochondrial proteins. Data were normalized to the VDAC or alpha-tubulin protein content and are expressed as fold increase over levels in the *TSC2*^+/+^ group. Open and closed bars represent *TSC2*^+/+^ and *TSC2*^-/-^ mice, respectively. Values represent the mean ± S.E.M. of data from 3–5 mice in each group. *P < 0.05 versus corresponding control. Scale bars: 2 micrometers in (A); 1 micrometer in insets of (A).

Mitochondria are the major source of energy production in cells and mTORC1 controls mitochondrial activity and biogenesis [33]. Therefore, we investigated the morphology of mitochondria using transmission electron microscopy. Ultrastructural analysis of *TSC2*^-/-^ hearts showed misalignment, aggregation and a decrease in size of mitochondria (Fig 4A), but the intra-mitochondrial structure appeared to be maintained, as shown in the image insets. The frequency of smaller mitochondria was significantly increased in *TSC2*^-/-^ hearts and the number of mitochondria per area was significantly bigger in *TSC2*^-/-^ hearts compared with *TSC2*^+/+^ hearts (Fig 4B). Dysfunctional mitochondria generate reactive oxygen species. We estimated the amount of HNE proteins, which is a marker for oxidative stress, in the mitochondrial fraction (Fig 4C). The amount was significantly increased in *TSC2*^-/-^ hearts, suggesting the accumulation of oxidative stress. However, PINK1 and Parkin, molecules related to mitophagy was decreased (Fig 4D) and total mitochondrial amount in the heart indicated by the levels of mitochondria-specific proteins, such as VDAC (a marker for outer membrane) and Timm23 (a marker for inner membrane) was not different between the two groups (Fig 4E).

### Protein synthesis and autophagic activities in 4-week-old *TSC2^-/-^* hearts

To investigate the mechanism underlying cardiac dysfunction and mitochondrial morphologic changes in *TSC2*^-/-^ mice, we examined cardiac phenotypes of the mice at an earlier time point, when the secondary effects caused by cardiac dysfunction were minimal. We selected 4 weeks of age to minimize the effects of rapid growth, although the mice had already showed cardiac dysfunction estimated by echocardiography on awake mice (Fig 1F). The wall thickness indicated by IVSd and LVPWd was not different between the two groups (Fig 1F) in contrast to the 4-month-old mice. The mortality at 4 weeks was 0% (Fig 1B). As we observed in 4-month-old *TSC2*^-/-^ mice, HW/BW was increased (Fig 1E), CSA was increased (Fig 2D), apoptosis was not changed (Fig 2F), the expression level of *Nppa* was increased and that of *Col1a2* was not changed (Fig 2H) in 4-week-old *TSC2*^-/-^ mice. The expression level of *Myh7* was not changed, while it was increased in 4-month-old *TSC2*^-/-^ mice. These data suggest the progression of cardiomyopathy in *TSC2*^-/-^ mice with time. In Western blot analysis, the phosphorylation level of Akt was significantly decreased and those of S6 and 4E-BP1 were significantly increased in the heart of *TSC2*^-/-^ mice compared with those of *TSC2*^+/+^ mice (Fig 3D). There was no significant difference in the level of KDEL or ubiquitinated proteins (Fig 3E). These are the same changes as observed in 4-month-old *TSC2*^-/-^ mice. In contrast to the 4-month-old hearts, the level of phosphorylated AMPK was significantly decreased in *TSC2*^-/-^ hearts, and the expression level of TSC1 showed no significant difference (Fig 3D). The protein level of p62 was significantly increased in *TSC2*^-/-^ hearts, as observed in 4-month-old *TSC2*^-/-^ mice. The protein level of LC3-II was increased (Fig 3F), while that showed no significant increase in 4-month-old *TSC2*^-/-^ mice. These suggest that the autophagic flux was attenuated due to impairment of autophagosome removal.

### Mitochondrial morphology and function in 4-week-old *TSC2^-/-^* hearts

**Fig 5:**
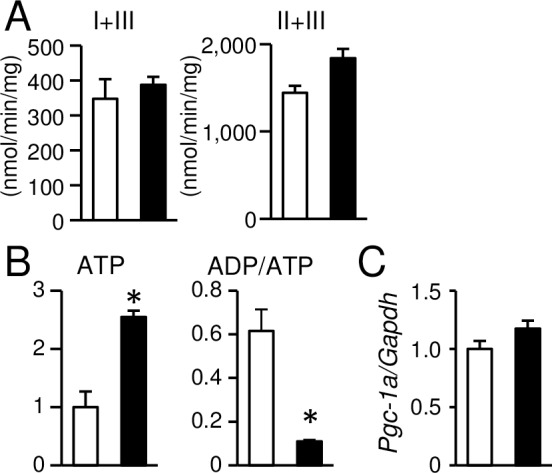
Biochemical analyses of mitochondrial function in *TSC2*^-/-^ hearts at 4 weeks of age. (A): The activities of complex I + III and complex II + III in mitochondria isolated from the hearts. (B): The ATP content expressed as fold increase over levels in the *TSC2*^+/+^ group and ADP to ATP ratio (ADP/ATP) in the heart tissues. (C): mRNA expression of *Pgc-1a* was determined by quantitative RT-PCR. Data were normalized to the *Gapdh* content and are expressed as fold increase over levels in the *TSC2*^+/+^ group. Values represent the mean ± S.E.M. of data from 3–4 mice in each group. Open and closed bars represent *TSC2*^+/+^ and *TSC2*^-/-^ mice, respectively. *P < 0.05 versus corresponding control.

Ultrastructural analysis revealed a decrease in size and an increase in number of mitochondria in 4-week-old *TSC2*^-/-^ hearts (Fig 4F and 4G), as observed in the 4-month-old hearts. As impaired mitochondrial function might be a cause for the cardiac dysfunction seen in *TSC2*^-/-^ mice, we examined mitochondrial function by measuring respiratory chain enzyme activity in mitochondria isolated from mouse hearts (Fig 5A). However, there was no significant difference in the activity of either complex I + III or complex II + III between the two groups. We estimated the amount of HNE proteins in the mitochondrial fraction (Fig 4H). There was no significant difference in the protein level between the two groups, although that was increased in 4-month-old *TSC2*^-/-^ mice. The expression levels of PINK1 and Parkin were decreased (Fig 4I) as observed in 4-month-old *TSC2*^-/-^ mice. Furthermore, we examined ATP concentration and the ratio of ADP to ATP concentration (ADP/ATP) in the heart tissue (Fig 5B). ATP concentration was found to be significantly increased in *TSC2*^-/-^ hearts compared with *TSC2*^+/+^ hearts, and a significant decrease in ADP/ATP was detected. The mRNA expression of *Pgc-1a* measured by quantitative RT-PCR was preserved in *TSC2*^-/-^ hearts (Fig 5C). These data indicate that the mitochondrial function was maintained in *TSC2*^-/-^ hearts, although mitochondrial morphology was changed. Thus, the main cause of the cardiac phenotype seen in *TSC2*^-/-^ hearts is not mitochondrial dysfunction.

### Attenuation of the cardiac phenotypes in TSC2-deficient hearts by upregulation of autophagic flux

**Fig 6:**
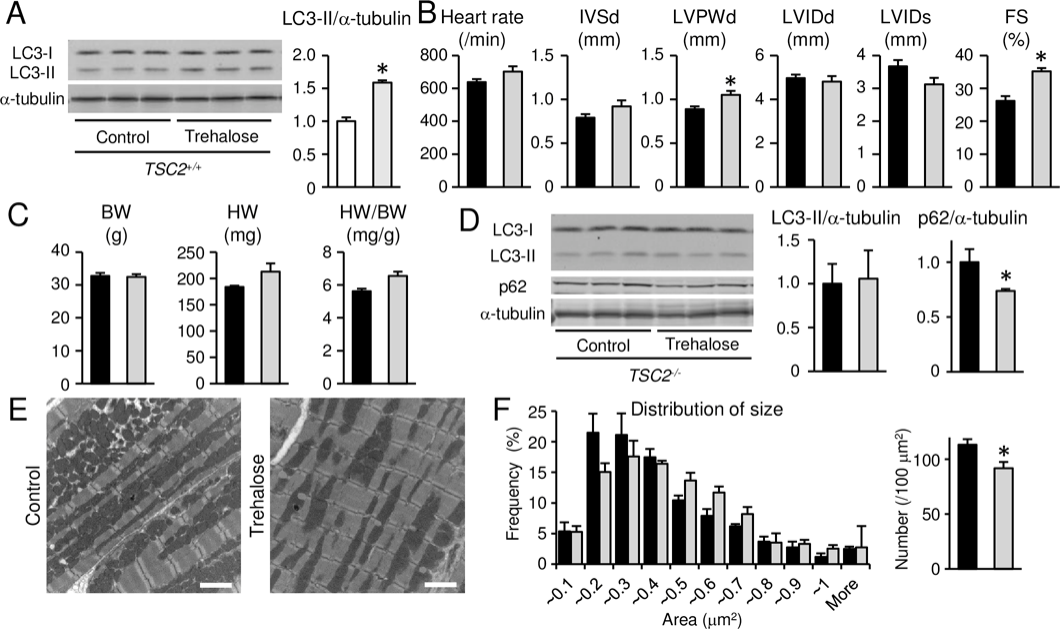
Effects of trehalose treatment on cardiac phenotypes in *TSC2*^-/-^ mice. (A): Western blot analysis for LC3 in trehalose-treated *TSC2*^*+/+*^ group. Data were normalized to alpha-tubulin protein content. White and light gray bars represent control *TSC2*^+/+^ mice and trehalose-treated *TSC2*^+/+^ mice, respectively. (B): Echocardiographic parameters of the awake *TSC2*^-/-^ mice. (C): Physiological parameters of the *TSC2*^-/-^ mice. BW, HW and HW/BW indicate body weight, whole heart weight and whole heart-to-body-weight ratio, respectively. (D): Western blot analysis for LC3 and p62. Data were normalized to alpha-tubulin protein content. All data are expressed as fold increase over levels in control *TSC2*^-/-^ group. (E and F): Electron micrographs of the *TSC2*^-/-^ hearts (E) and quantitative analyses of mitochondrial size and number (F). In (B) to (F), black and dark gray bars represent control *TSC2*^-/-^ mice and trehalose-treated *TSC2*^-/-^ mice, respectively. Values represent the mean ± S.E.M. of data from 3 to 5 mice in each group. *P < 0.05 versus control group. Scale bars: 2 micrometers.

We hypothesized that reduced autophagic flux is one of the mechanisms for the cardiac phenotypes seen in *TSC2*^-/-^ mice. To investigate the involvement of autophagy in cardiac phenotypes in *TSC2*^-/-^ hearts, we treated the *TSC2*^-/-^ mice with trehalose. Trehalose is a non-reducing disaccharide found in a wide variety of organisms, including bacteria, yeast, invertebrates, and plants and functions to protect cells against various environmental stresses [34]. Recently, it is reported that trehalose acts as an mTOR-independent autophagy activator and can reduce protein aggregates in some mouse disease models [35–37]. First, we treated *TSC2*^+/+^ mice with trehalose for 10 weeks starting from 6 weeks after birth to confirm upregulation of autophagic activity. Western blot analysis for LC3 indicated that autophagy was upregulated (Fig 6A). Thus, we treated *TSC2*^-/-^ mice with trehalose for 10 weeks starting from 6 weeks after birth. After the treatment, cardiac function indicated by echocardiographic fractional shortening was significantly improved in the trehalose-treated group compared with control non-treated group (Fig 6B). The heart weight and the ratio of heart to body weight were not different between the two groups (Fig 6C). The accumulation of p62 was significantly reduced in trehalose-treated *TSC2*^-/-^ hearts compared with control *TSC2*^-/-^ hearts, although the protein level of LC3-II was not different (Fig 6D). This supports that trehalose increased autophagic flux in *TSC2*^-/-^ mice. Ultrastructural analysis showed that the size of mitochondria was larger and the number was smaller in trehalose-treated group than those in control group (Fig 6E and 6F).

## Discussion

In this study, we showed that the TSC-mTORC1 signaling pathway plays an important role to maintain cardiac function and to regulate mitochondrial size and number. Its homeostatic function is mediated through autophagy. Since mitochondria were not damaged in *TSC2*^-/-^ hearts, autophagy is the main player in this system rather than mitochondria-specific autophagy, mitophagy.

It has been reported that cardiac-specific TSC1-deficient mice, which were generated by crossing floxed *TSC1* mice with myosin light chain 2v-promotor driven *Cre* recombinase knock-in mice, had a median survival of 6 months and developed dilated cardiomyopathy [38]. Since TSC1 is required to stabilize TSC2 and prevents its ubiquitin-mediated degradation [29, 30], ablation of TSC1 would lead to downregulation of TSC2. It is reasonable that TSC1-deficient mice developed similar cardiac phenotypes to *TSC2*^-/-^ mice. Thus, the TSC1/2 complex is essential for maintenance of cardiac function after birth, but not for heart development or survival during embryonic stage. Although the molecular mechanisms underlying the development of dilated cardiomyopathy in the TSC1-deficient mice have not been investigated, histological analysis of the mice showed the occurrence of scattered foci of enlarged cardiomyocytes accumulating glycogen without any evidence of proliferation in the lesion [38]. However, we did not observe such histological changes nor tumorigenesis in *TSC2*^-/-^ hearts macroscopically and by echocardiography. Although the molecular mechanism underlying the difference in the histological findings between cardiac-specific TSC1 and TSC2-deficient hearts is unknown, it may be due to the timing of gene knockdown or distinct functions of the products of these genes.

*TSC2* ablation in the heart led to not only cardiac dysfunction, but also a decrease in heart rate. ATP is released from cardiomyocytes [39, 40] and immediately metabolized to adenosine by ATPases that have their catalytic domain on the outer side of plasma membrane. Since adenosine is known to have a negative effect on heart rate [41], increased cytosolic ATP level may result in a decrease in heart rate in *TSC2*^-/-^ mice. Other possibilities are that histological abnormalities including misalignment of mitochondria or direct effects of the TSC2 signaling pathway impair the function of the conduction system in the heart.

Autophagic flux indicated by protein level of LC3-II and p62 was decreased in *TSC2*^-/-^ hearts at 4 weeks of age. Co-existence of increased LC3-II with increased p62 protein levels is consistent with impaired autophagosome removal rather than decreased formation. mTORC1 has been reported to regulate autophagy by signalling to ULK1 and lysosome homeostasis [5, 42].

We observed cardiomyocyte hypertrophy indicated by increases in heart-to-body weight ratio, LV mass and cross-sectional area of cardiomyocytes in *TSC2*^-/-^ mice. Absence of fibrosis and cardiomyocyte death indicates that cardiac remodeling observed in *TSC2*^-/-^ mice is not typical for general dilated cardiomyopathy. The hypertrophic responses can be induced by activation of protein synthesis. We previously reported that inhibition of autophagy in cardiomyocytes leads to cardiac hypertrophy [7]. In this study, as the treatment with trehalose had no effect on cardiac hypertrophy, the results suggest that inhibition of autophagy is not involved in the development of cardiac hypertrophy in *TSC2*^-/-^ mice.

Our previous results showed that inhibition of autophagy in the heart by ablation of *Atg5* induced misalignment and heterogeneous size of mitochondria and damage of intramitochondrial structure, impaired mitochondrial function and increased oxidative stress [7, 28]. If we consider the importance of autophagy for maintenance of normal functional mitochondria, it is surprising that *TSC2*^-/-^ hearts showed normal mitochondrial respiratory function even with alteration in mitochondrial number and size. The normal mitochondrial function was confirmed by the results that ATP content was not decreased and oxidative stress was not increased in *TSC2*^-/-^ hearts. This is in agreement with the previously reported *in vitro* studies that mitochondrial activity is stimulated and ATP levels are increased under the condition of constitutive mTORC1 activation in TSC2-deficient or knockdown cells [33, 43, 44]. The TSC2/mTORC1-dependent autophagy pathway may not be required for the removal of damaged mitochondria at least in the absence of TSC2. However, the mitochondrial function was measured using isolated mitochondria in this study and the assay has experimental limitation. It has been reported that isolation of mitochondria causes the difference in functional characteristics compared to intact mitochondria in permeabilized miofibers [45]. mTORC1 is reported to control mitochondrial ATP production capacity by selectively promoting translation of nucleus-encoded mitochondria-related mRNA [33]. Hyperactivation of mTORC1 may protect mitochondria against stress. We observed an increase in number and a decrease in size of mitochondria in *TSC2*^-/-^ hearts. Consistent with our findings, the number of mitochondria is increased in pancreatic beta-cells lacking *TSC2* [44]. The quantity, quality and size of mitochondria are determined by fusion and fission, followed by autophagy [46]. It is possible that the TSC2/mTORC1-dependent signaling pathway may be directly or indirectly involved in mitochondrial dynamics.

The activation of AMPK leads to TSC2 phosphorylation, resulting in restoration of ATP through regulation of metabolism and inhibition of growth via the mTORC1 pathway. AMPK phosphorylation was impaired in our *TSC2*^-/-^ hearts, conversely the level was reported to be increased in tumor-derived cells or neurons lacking *TSC2* [47, 48]. The phosphorylation level of AMPK in *TSC2*^-/-^ hearts may be affected by the increase in intracellular ATP concentration. AMPK activates autophagy not only by inhibition of mTORC1 but also by direct phosphorylation of ULK1, which is required for autophagy induction [5, 48, 49]. In addition to activation of mTORC1 activity, inhibition of AMPK activity may supress autophagic activity in *TSC2*^-/-^ hearts. Although the decreased Akt phosphorylation level in *TSC2*^-/-^ hearts can inhibit mTORC1 via proline-rich Akt substrate of 40 kDa (PRAS40), an endogenous mTORC1 inhibitor, the negative feedback mechanism would not be enough for the normalization of the mTORC1 signaling pathway.

mTORC1 positively regulates cell growth and proliferation by promoting biosynthesis of proteins and lipids and mitochondrial metabolism and biogenesis and also by limiting autophagy [1]. Over-activating these functions of mTORC1 could be related to the observed phenotypes in *TSC2*^-/-^ hearts. mTOR controls mitochondrial oxidative function through a yin yang 1‒PGC-1alpha transcriptional complex [3]. PGC-1alpha is known to promote mitochondrial biogenesis and function. In *TSC2*^-/-^ hearts, mitochondrial function was maintained, ATP concentration was increased and the expression of PGC-1alpha was preserved. Therefore, we can exclude the possibility that impairment of mitochondrial metabolism and biogenesis is a cause for cardiac dysfunction in *TSC2*^-/-^ mice.

We previously reported that inhibition of autophagy by cardiac-specific Atg5-deficient mice induces age-related cardiomyopathy [28] and it was found that the accumulation and aggregation of damaged or unnecessary proteins could cause the malfunction of biological processes and play an important role in aging. As signaling pathways towards protein synthesis were significantly accelerated in *TSC2*^-/-^ hearts, there is a possibility that unnecessary or misfolded proteins were accumulated in cardiomyocytes to induce cardiac dysfunction. However, we observed no increase in ubiquitinated or KDEL proteins in *TSC2*^-/-^ hearts. Some compensatory mechanisms, such as TSC2/mTORC1-independent autophagy, chaperone-mediated autophagy and proteasome system, could be involved in the maintenance of protein turnover. It is also possible that the ability of mTORC1-dependent autophagy still might be enough to respond to the increased protein load.

PINK1 and Parkin are known to be key molecules for damaged mitochondria clearance via ubiquitination and mitophagy in neuron [50]. Surprisingly, the expression level of PINK1 and Parkin was reduced in *TSC2*^-/-^ hearts, suggesting PINK1 and Parkin-related mitophagy is down-regulated. Although it was reported that suppression of mTOR pathway can increase the expression level of PINK1 and Parkin [51], it is unclear why PINK1 and Parkin are reduced in this model. Further study is requested to clarify the effect of the decreases in PINK1/Parkin levels on the cardiac phenotype and mitochondrial morphology observed in *TSC2*^-/-^ mice.

The protein level of p62 was significantly decreased by treatment of trehalose for 10 weeks, suggesting that autophagic flux was improved by trehalose treatment. However, the protein level of LC3-II was not different between control and trehalose-treated *TSC2*^-/-^ groups. This might be due to the balance between the upregulation of autophagic induction and autolysosomal removal. The protective effect of trehalose on cardiac dysfunction in *TSC2*^-/-^ mice indicates the existence of an mTOR-independent pathway to enhance autophagic flux. Upregulation of autophagic activity by trehalose treatment improved the cardiac function, indicating that downregulation of autophagy could be a significant cause for cardiac phenotypes in *TSC2*^-/-^ mice. Since the extent of rescue of the cardiac phenotypes was partial, we could not exclude possibilities that mechanisms other than autophagy are involved in the development of cardiac phenotypes in *TSC2*^-/-^ mice. Trehalose is not a selective activator of autophagy [34]. Furthermore, trehalose does not appear to be absorbed and is rather broken down to glucose in the gastrointestinal tract of mammals [52]. Thus, we cannot exclude a possibility that improvement in cardiac function is due to the other potential “off-target” effects of trehalose. Molecular mechanisms how downregulation of autophagic flux in *TSC2*^-/-^ mice resulted in the impairment of cardiac function remain to be elucidated. Cytoarchitectural disorganization would lead to cardiac dysfunction [53]. Ultrastructural images of *TSC2*^-/-^ hearts showed misalignment of mitochondria and looked as if many of mitochondria could not interact with myofibril closely, suggesting that energy transfer between mitochondria and myosin-ATPase could be restricted. Activated autophagy can remove not only small mitochondria but also other cytoplasmic components and improve the interaction and energy transfer between mitochondria and myofibril. It is also possible that the misaligned mitochondria simply disturb sarcomere contraction.

In conclusion, we demonstrated that constitutive stimulation of mTORC1 signaling by *TSC2* ablation in the heart leads to cardiac chamber dilatation and dysfunction and that upregulation of autophagic activity attenuates the cardiac dysfunction. Autophagy regulated by TSC-mTORC1 signaling in the heart is essential for the control of mitochondrial quantity. It is reported that mTORC1 inhibitors such as rapamycin can be pharmacological treatments of TSC disease [54, 55]. Trehalose could be a novel therapeutic for TSC patients. However, further investigation is required to reveal the involvement of autophagy in the pathogenesis of the disease with benign tumours.

## Supporting Information

S1 Fig Echocardiographic analysis of the mice at 4 months of age under anaesthesia. (PDF)

S2 Fig The original blots shown in Figs 1, 3, 4 and 6. (PDF)

S1 Protocol Methods for the pilot study. (DOCX)
