# Supplementary material for: mTOR Hyperactivation by Ablation of Tuberous Sclerosis Complex 2 in the Mouse Heart Induces Cardiac Dysfunction with the Increased Number of Small Mitochondria Mediated through the Down-Regulation of Autophagy
Source: PLoS One. 2016 Mar 29;11(3):e0152628. doi: 10.1371/journal.pone.0152628 (PMC4811538; doi:10.1371/journal.pone.0152628)
Supplement: S1 Fig — (PDF) [file pone.0152628.s001.pdf]

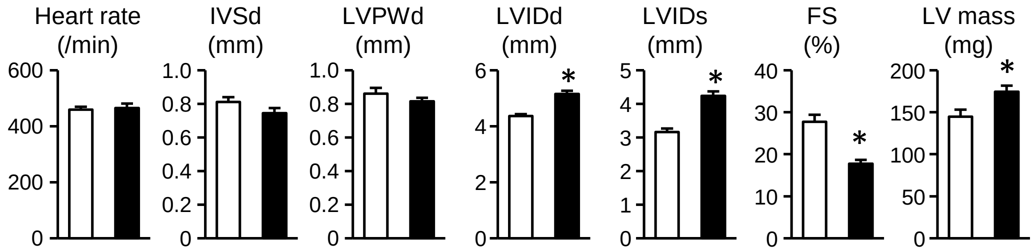

S1 Fig. Echocardiographic analysis of the mice at 4 months of age under anaesthesia

Open and closed bars represent *TSC2*$^{+/+}$ and *TSC2*$^{-/-}$ mice, respectively. Values represent the mean ± S.E.M. of data from 5 mice in each group. *$P < 0.05$.
