# Supplementary material for: mTOR Hyperactivation by Ablation of Tuberous Sclerosis Complex 2 in the Mouse Heart Induces Cardiac Dysfunction with the Increased Number of Small Mitochondria Mediated through the Down-Regulation of Autophagy
Source: PLoS One. 2016 Mar 29;11(3):e0152628. doi: 10.1371/journal.pone.0152628 (PMC4811538; doi:10.1371/journal.pone.0152628)
Supplement: S2 Fig — (PDF) [file pone.0152628.s002.pdf]

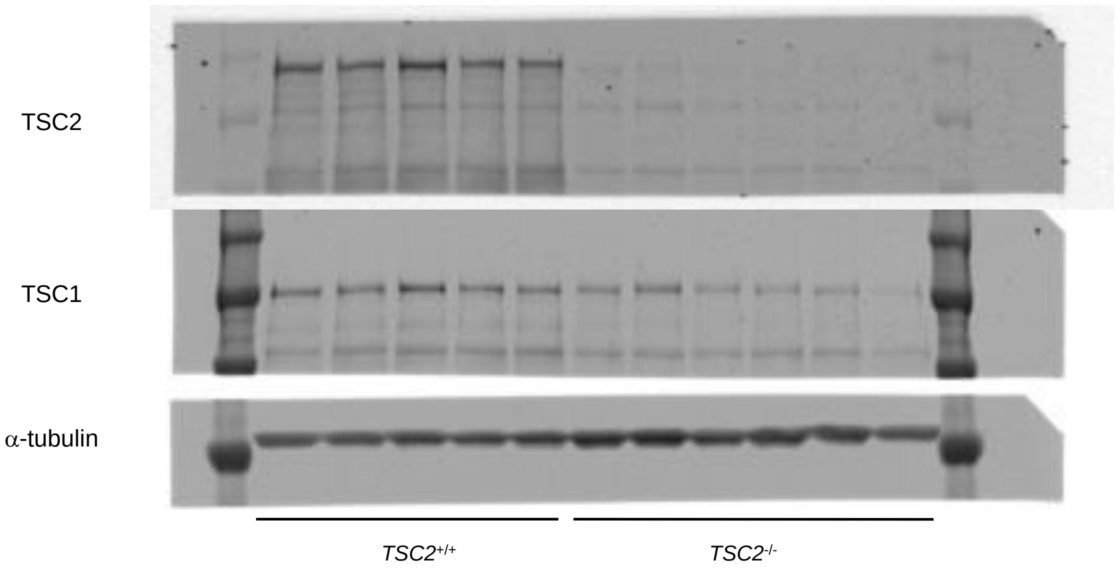


**S2A Fig. The original blots shown in Fig. 1.**

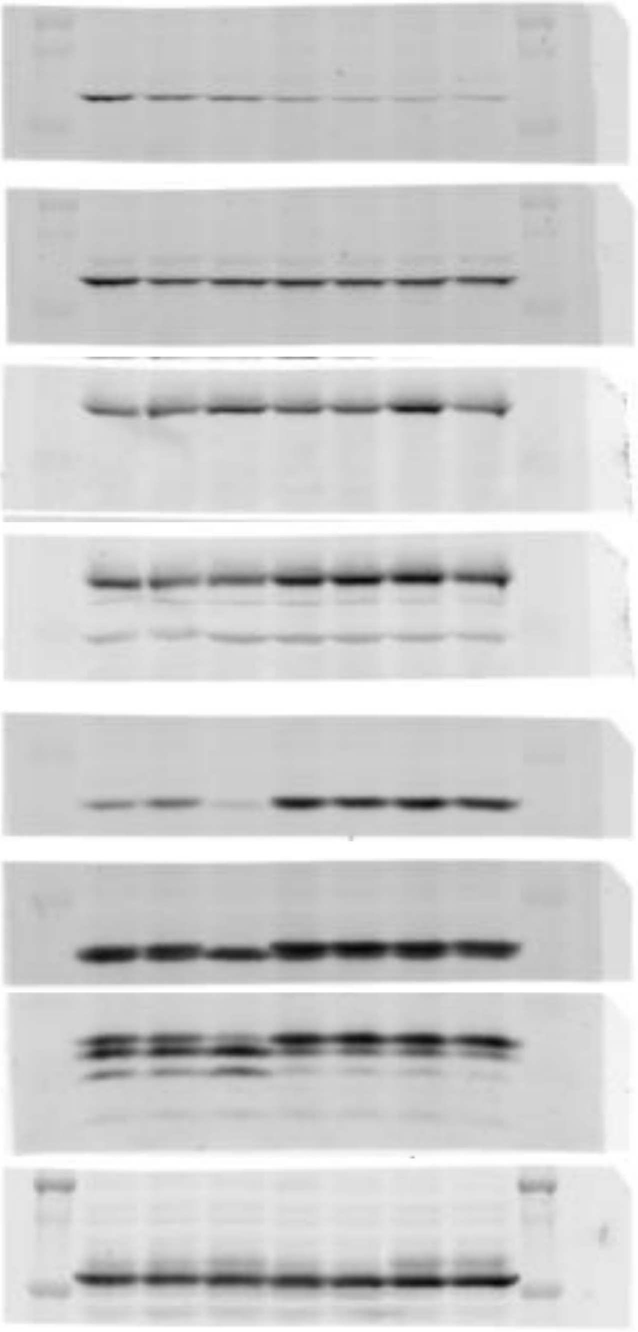


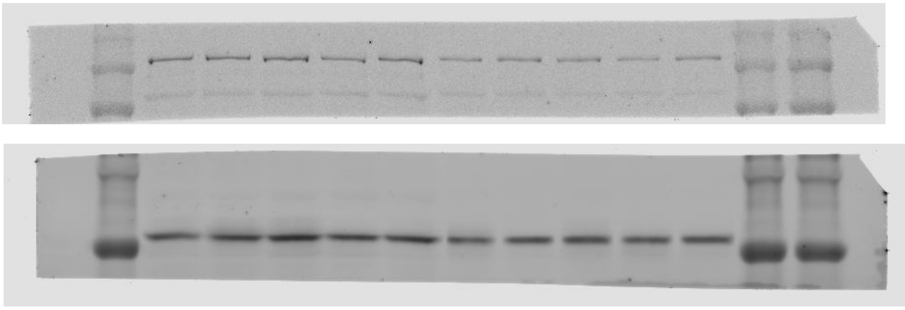


**S2B Fig. The original blots shown in Fig. 3A.**

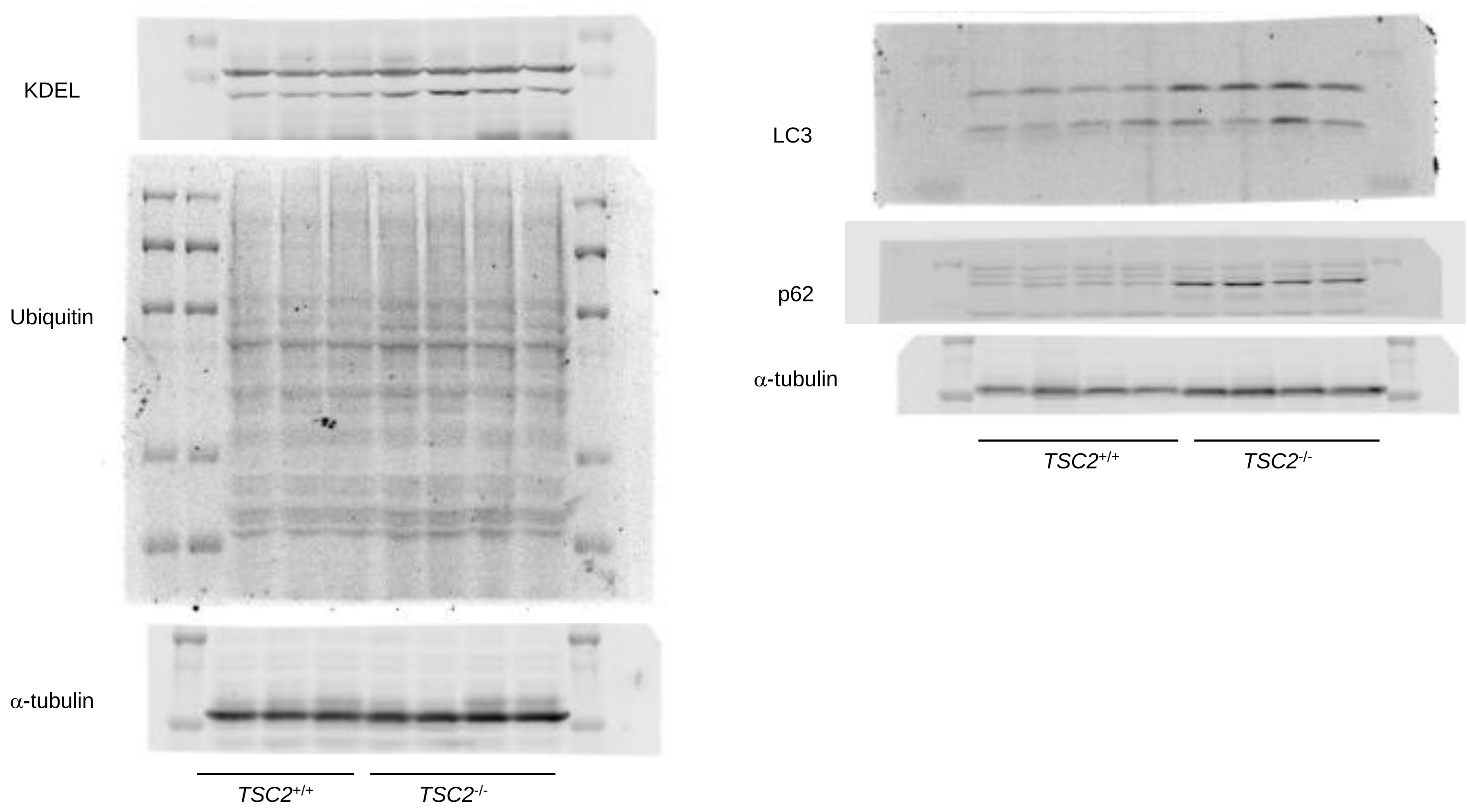


**S2C Fig. The original blots shown in Fig. 3B and C.**

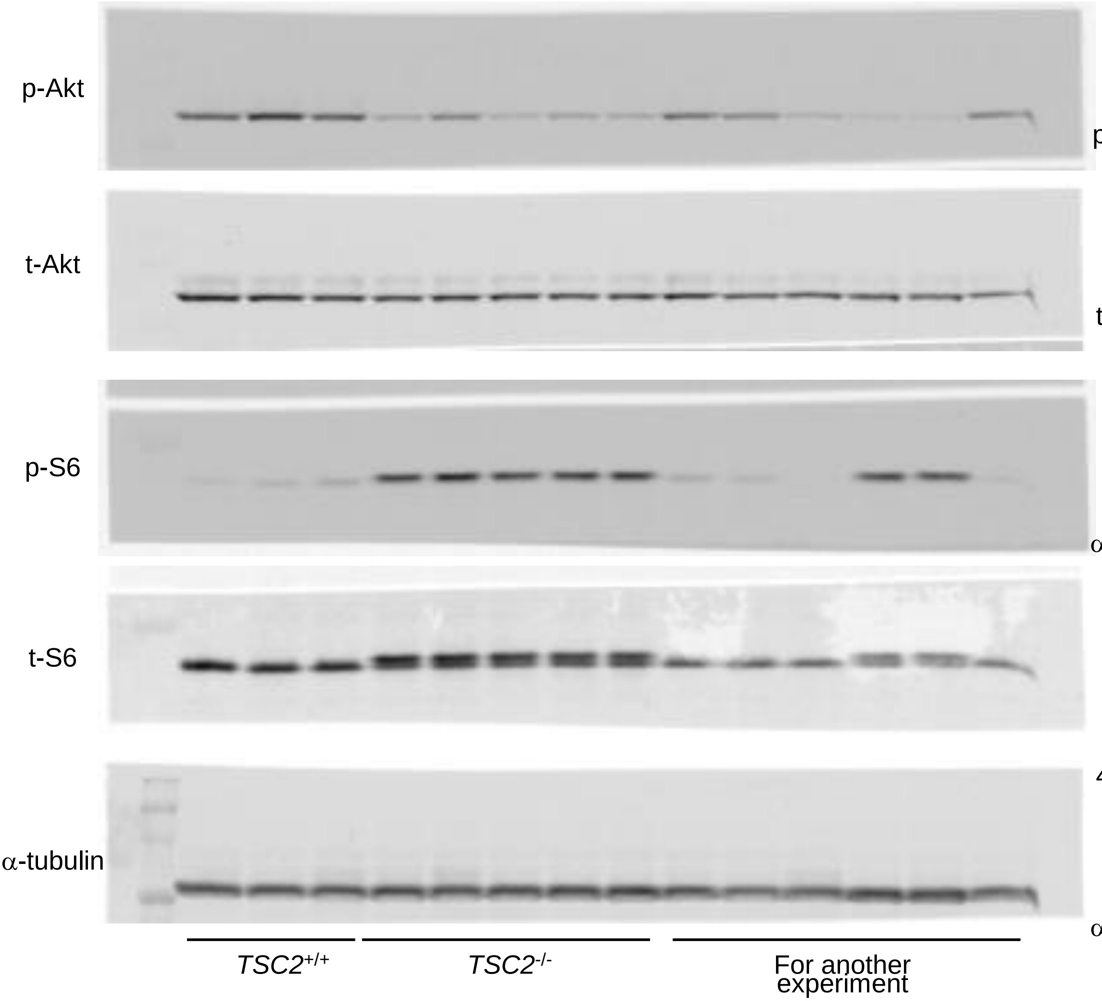


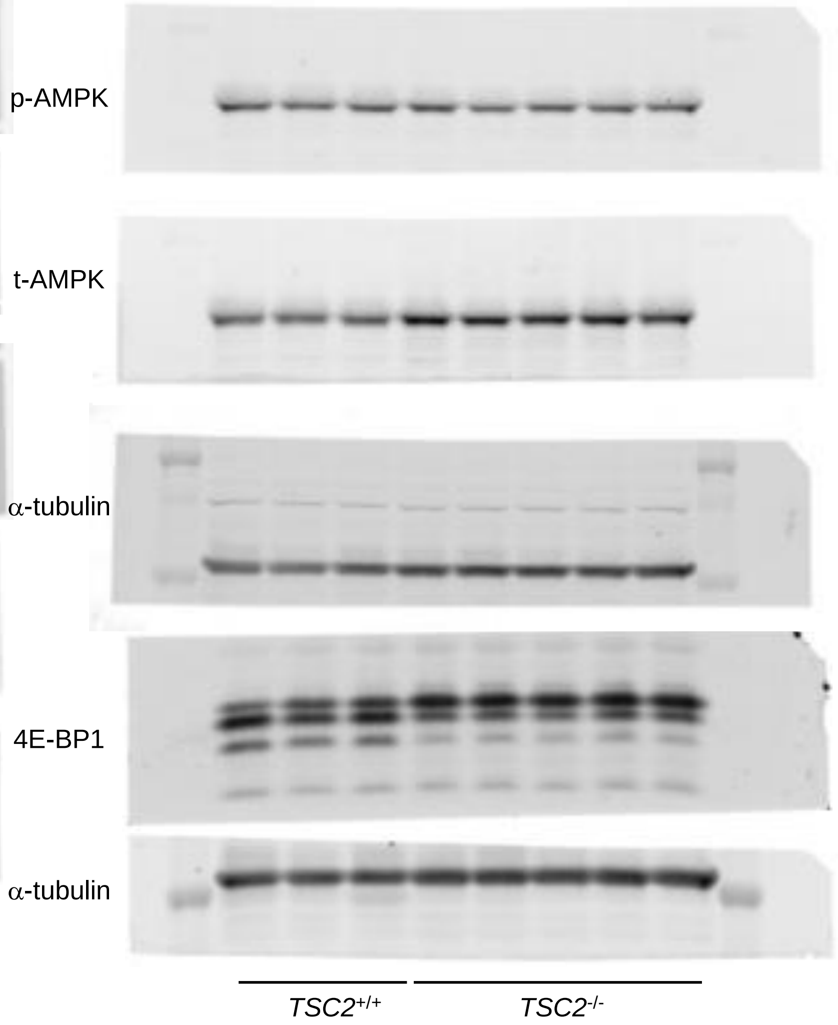


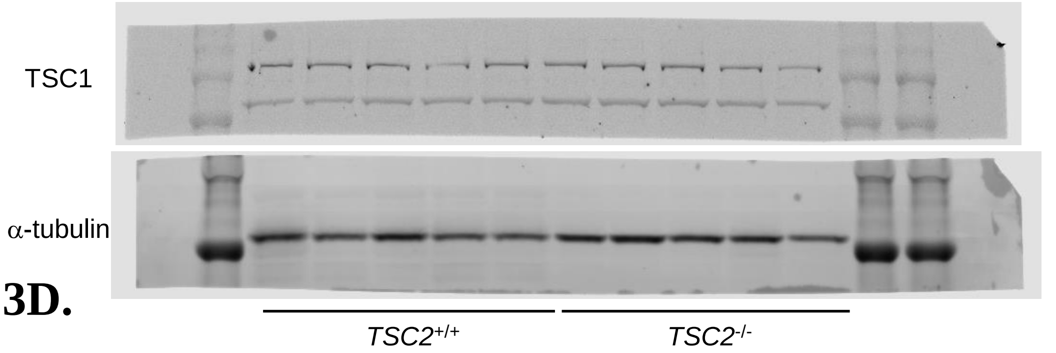


**S2D Fig. The original blots shown in Fig. 3D.**

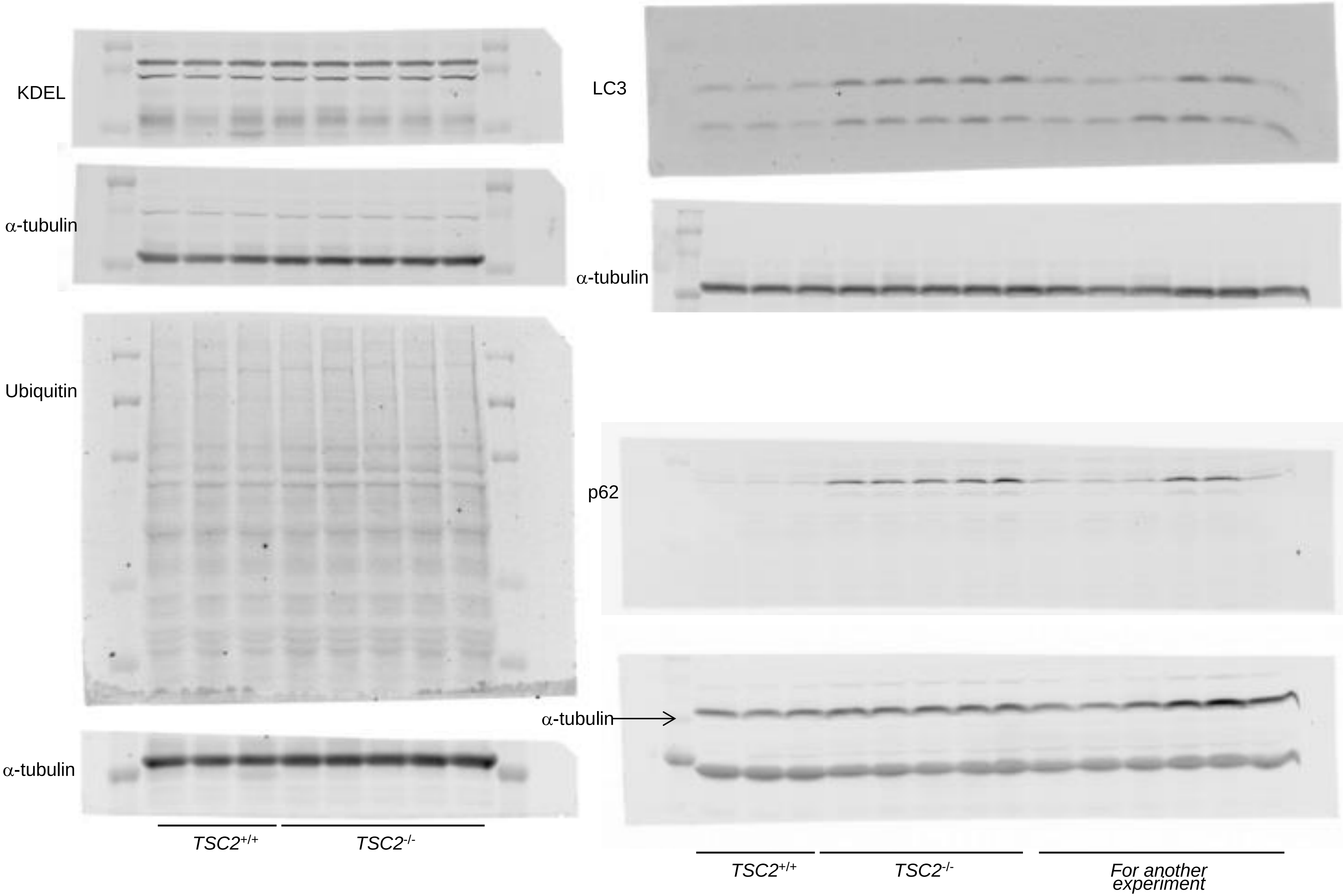


**S2E Fig. The original blots shown in Fig. 3E and F.**

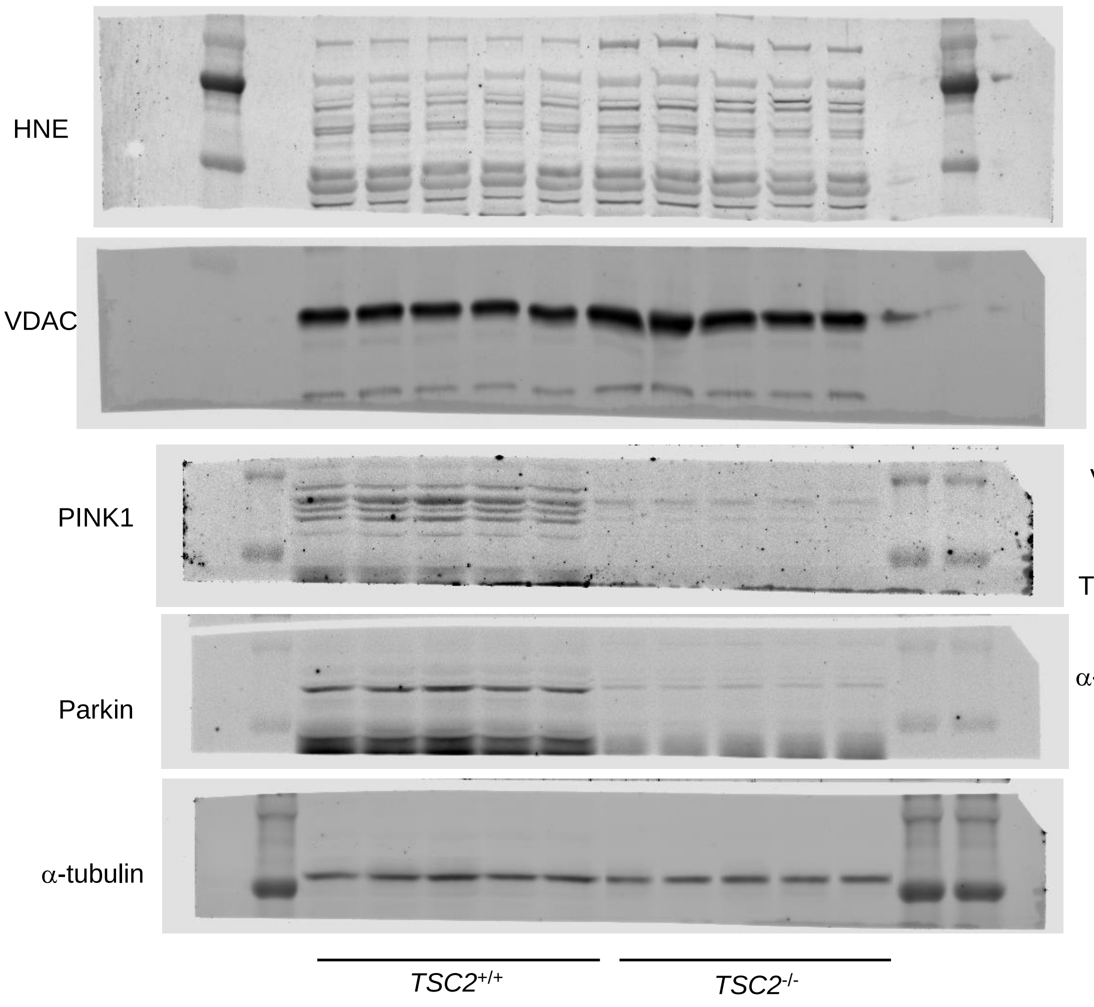


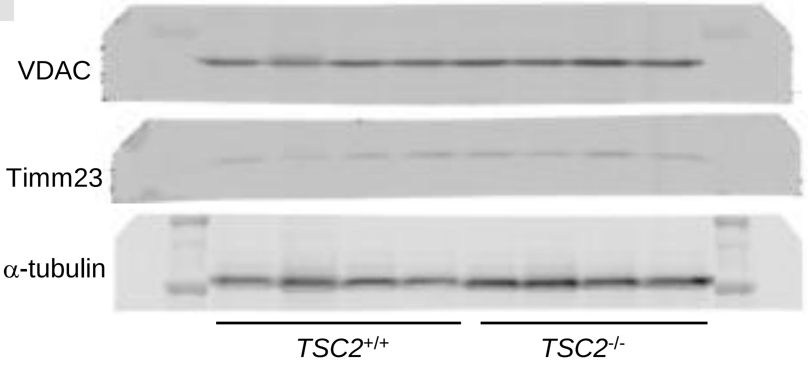


**S2F Fig. The original blots shown in Fig. 4C, D and E.**

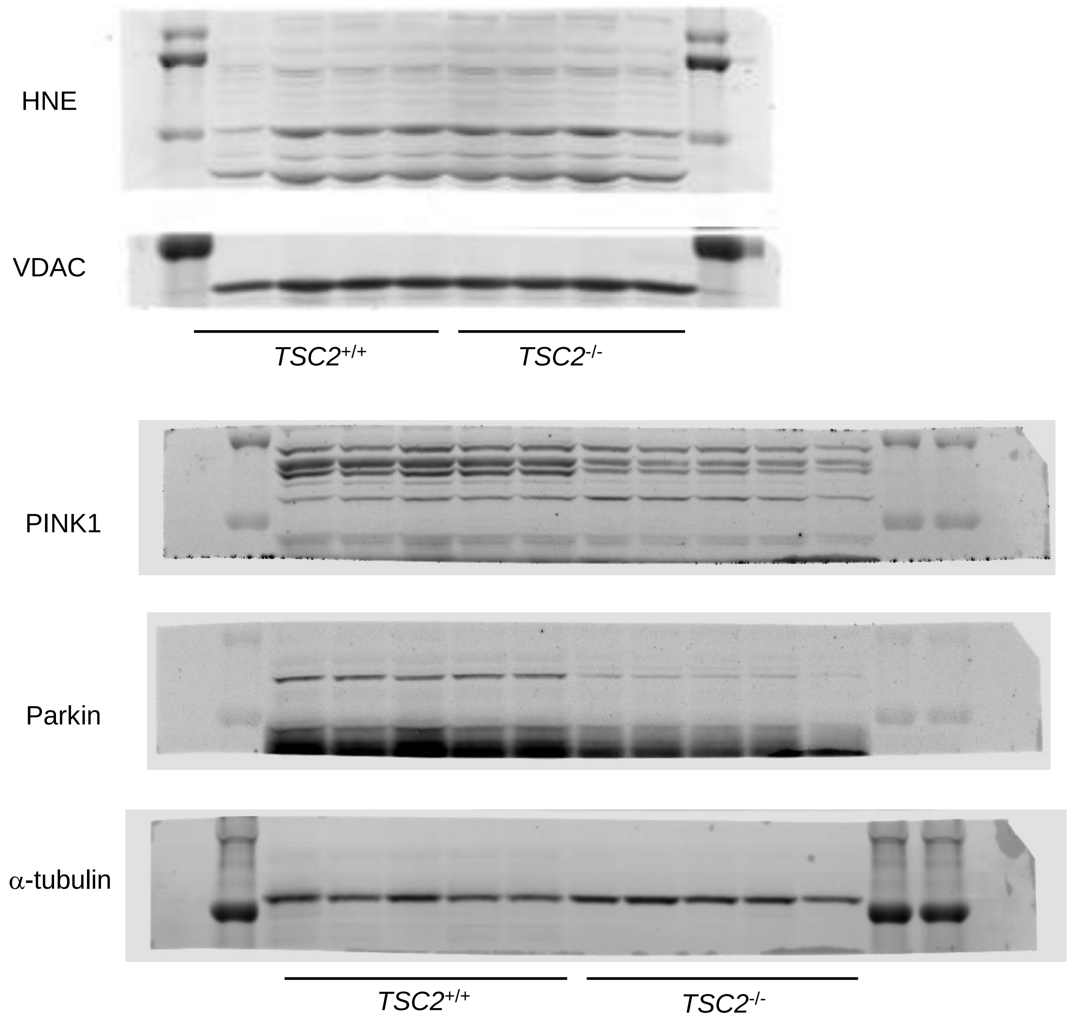


**S2G Fig. The original blots shown in Fig. 4H and I.**

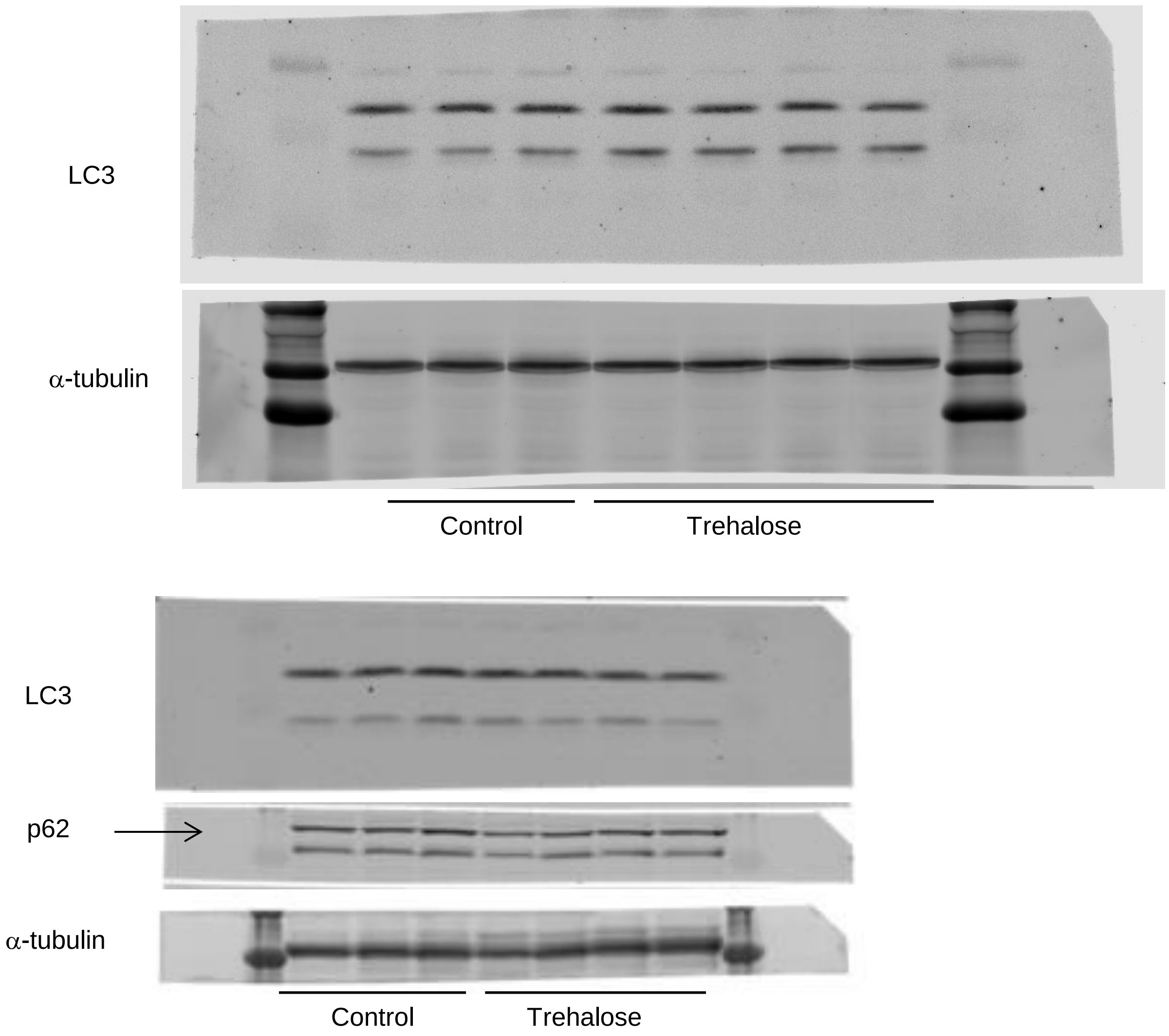


**S2H Fig. The original blots shown in Fig. 6A and D.**
