# Supplementary material for: mTOR Hyperactivation by Ablation of Tuberous Sclerosis Complex 2 in the Mouse Heart Induces Cardiac Dysfunction with the Increased Number of Small Mitochondria Mediated through the Down-Regulation of Autophagy
Source: PLoS One. 2016 Mar 29;11(3):e0152628. doi: 10.1371/journal.pone.0152628 (PMC4811538; doi:10.1371/journal.pone.0152628)
Supplement: S1 Protocol — (DOCX) [file pone.0152628.s003.docx]

**S Protocol. Methods for the pilot study**

The aim of the pilot study was to investigate whether the TSC2 deficiency causes not only cardiac contractile dysfunction but also pulmonary congestion and cardiac death, which are characteristics of human heart failure. The final goal of our study is to develop new therapeutics to treat patients with heart failure.

We used 8 of *TSC2*^-/-^ mice in the pilot study. The genetic backgrounds of the *TSC2*^flox/flox^ and α-MyHC-*Cre* mice are 129/SvJ x C57B/6J and C57B/6J, respectively.

In our pilot study design, *TSC2*^-/-^ mice are followed for 1 year with daily monitoring and any animals that show signs of significant clinical cardio-respiratory distress (rapid breathing, wheezing) or other serious clinical problems (marked reduction in activity and significant weight loss) are closely monitored at frequent intervals. Careful attention is paid to heating, body weight, hydration, and signs of distress. The mice are culled within 24 hours in case that there is no improvement.

In our actual pilot study, all mice died within 24 hours after the poor clinical conditions were observed or suddenly died without showing any signs of abnormal symptoms. The number of unexpected deaths in the pilot study was 4. The mice died between 4 to 8 months of age.
